# Data set on performance evaluation of discharged wastewater quality from Guna water treatment plant for potable water purpose

**DOI:** 10.1016/j.dib.2019.104926

**Published:** 2019-12-04

**Authors:** Adane Adugna Ayalew, Tayachew Takele

**Affiliations:** Faculty of Chemical and Food Engineering Bahir Dar Institute of Technology, Bahir Dar University Bahir Dar, Ethiopia

**Keywords:** Bottled drinking water, Discharged water, Potable water, Water treatment, WHO standards

## Abstract

This article provides to evaluate the quality of discharged water from Guna spring water treatment plant and to compare with WHO drinking water standards and Ethiopian bottled drinking water specification to reuse for potable water purpose. The discharged waste water quality analysis was conducted by Physical, Chemical, Biological and Bacteriological parameters of water in association with set of standards. Atomic adsorption spectroscopy (AAS), Flame photometer, UV–Visible spectrometer and Photo meter were used for characterization discharged water. All water quality parameter was determined in all unit process (softener, sand filter, activated carbon and ultra-filter). Bacteriological analysis (total coli form, fecal coli form and *Escherichia coli* type1) were conducted. During discharge water quality analysis, the effect of flow rate (2 m^3^/h, 4 m^3^/h and 6 m^3^/h) and discharge time (5,10 and 15 min) have been performed. We provided data set about Atomic adsorption spectroscopy (AAS), Flame photometer, Turbidity meter, Photometer and Bacteriological analysis parameter were verified.

**Table:** 

Subject	Environmental Science
Specific subject area	Water quality, drinking water
Type of data	Table Figure
How data were acquired	UV–Visible spectrometer, AAS, Flame photometer, turbid metre, Photo meter
Data format	Raw and Processed data
Experimental Parameters	Three flow rate (2,4 and 6 m^3^/hr) Three discharge time (5,10 and 15 min) Four-unit process (softener, sand filter, activated carbon and ultra filter) Two washing type (Back wash and surface wash) and also at diffrent sampling point (raw water, before ultra filter, ultra filter, treament plant, and reused water) were analyzed.
Data source location	Institution: Bahir Dar Institute of Technology City/Town/Region: Debre Tabor and Bhir Dar Country: Ethiopia Latitude and longitude; and GPS coordinates 11.5742^o^N, 37.3614^o^E and 11°35′37.10″N 37°23′26.77″E respectively for Bahir Dar. Latitude and longitude; and GPS coordinate 11.8567^o^N, 38.0155^o^E and 38° 0′ 55.8792″ Erespectively for Debre Tabor.
Data accessibility	Repository Name: [Medley Data] Data identification number**:** DOI: 10.17632/6j69bc2mdx.2 Direct URL to data: https://doi.org/10.17632/6j69bc2mdx.2

**Table:** 

**Value of the Data** • Determining and analysing of the physico-chemical and biological properties of discharged Guna spring water treatment plant as potable water purpose is very significant to the factory and to the local community user. Data of water analysis parameter are used to know about the discharged waste water as potential for drinking purpose and other mini application by recycling the discharge water. • The data set could be use as preliminary data for any engineers who want to design water treatment system and to redesign the discharged waste water for each unit operation and process unit for reuse. Researcher can use this data as raw pre-experiment for insight in water treatment system. • In general, all data set can be used for designing water treatment plant and waste water treatment system for any waste water treatment plant.

## 1 Data

The data set in this article reports on number of physical, biological, chemical and bacteriological property of Guna spring discharge water data set of the two-washing system (Back wash and surface wash) at different filter process (sand filter, softener, activated carbon and ultra-filter), and different sampling point (raw water, before ultra filter, ultra-filter, treatment plant and reused water) with different operational factors. Six kinds of raw data for water quality analysis were reported from mendeley data. Physico-chemical analysis on different parameters and unit operation such as sand, activated carbon, softener and ultra-filter data set, Mean value of nitrate and phosphate data set, Physico-chemical analysis at different sampling point data set, Mean value of bacteriological analysis data set, Physico-chemical analysis different sampling with different seasonal data set and Physico-chemical analysis of discharged water mixed with nearest River data set are present in Tables from micro excel file.

**Fig. 1:**
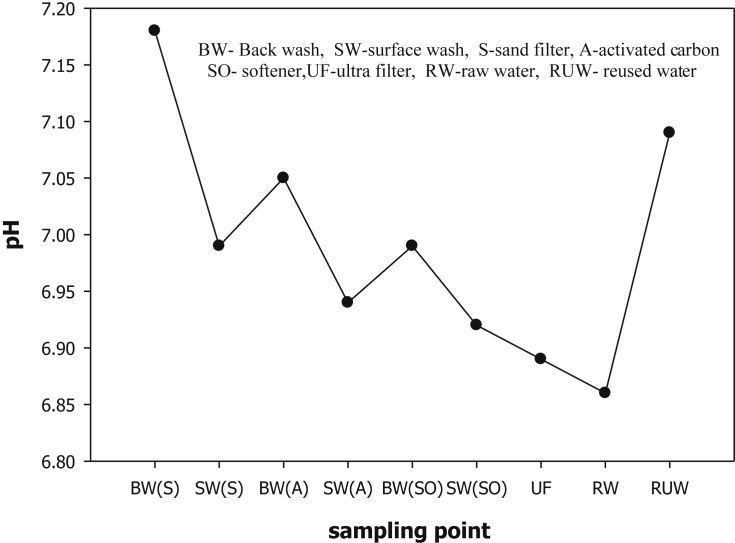
Average pH value measured at different sampling points.

**Fig. 2:**
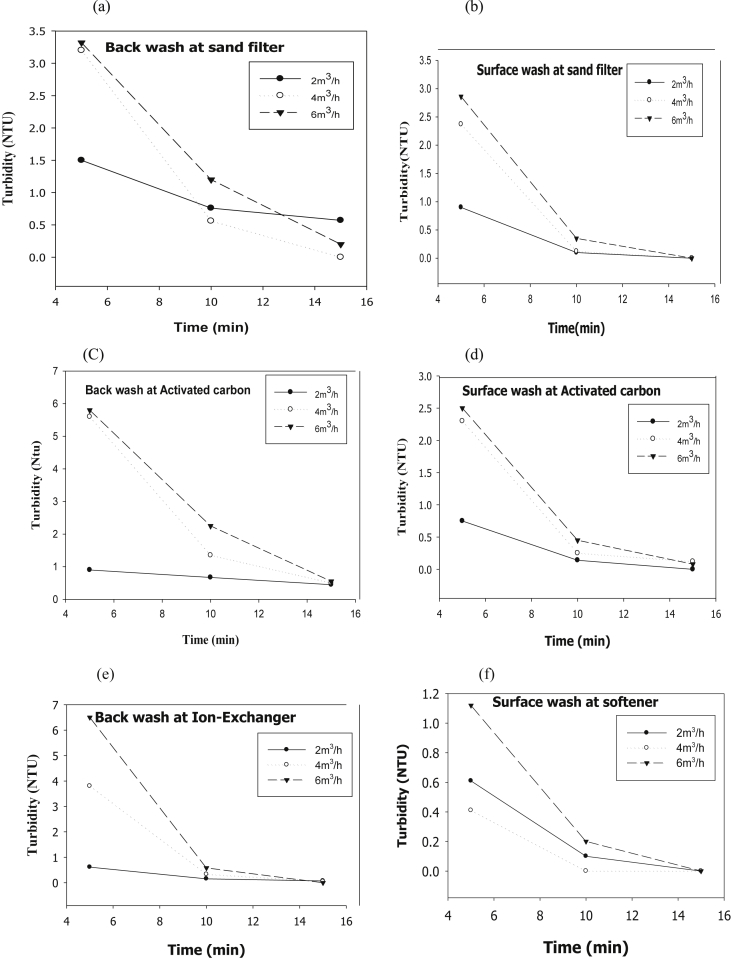
Turbidity value at (a) sand filter BW and (b) SW (c) activated Carbon at BW and (d) SW (e) ion-exchanger at BW and (f) SW.

**Fig. 3:**
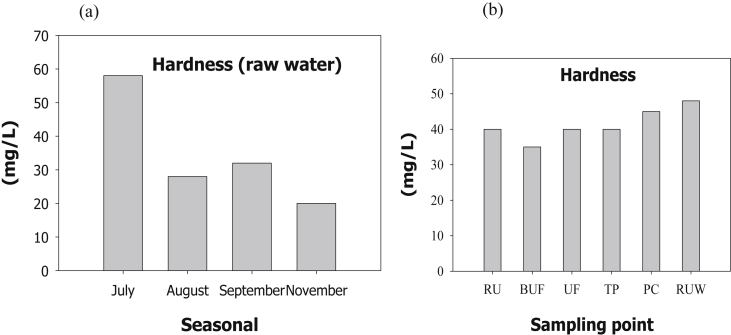
Total hardness of (a) RW at different seasonal and (b) different sampling point.

**Fig. 4:**
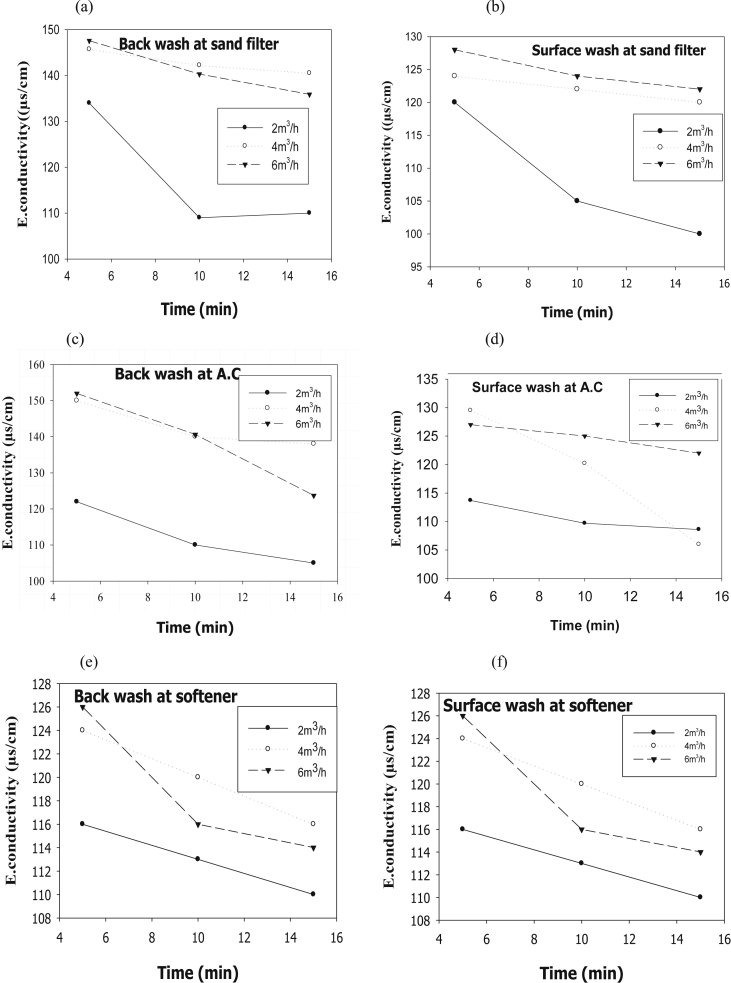
E. conductivity value at (a) sand filter BW and (b) SW, (c) activated carbon at BW and (d) SW, (e) ion-exchanger at BW and (f) SW.

**Fig. 5:**
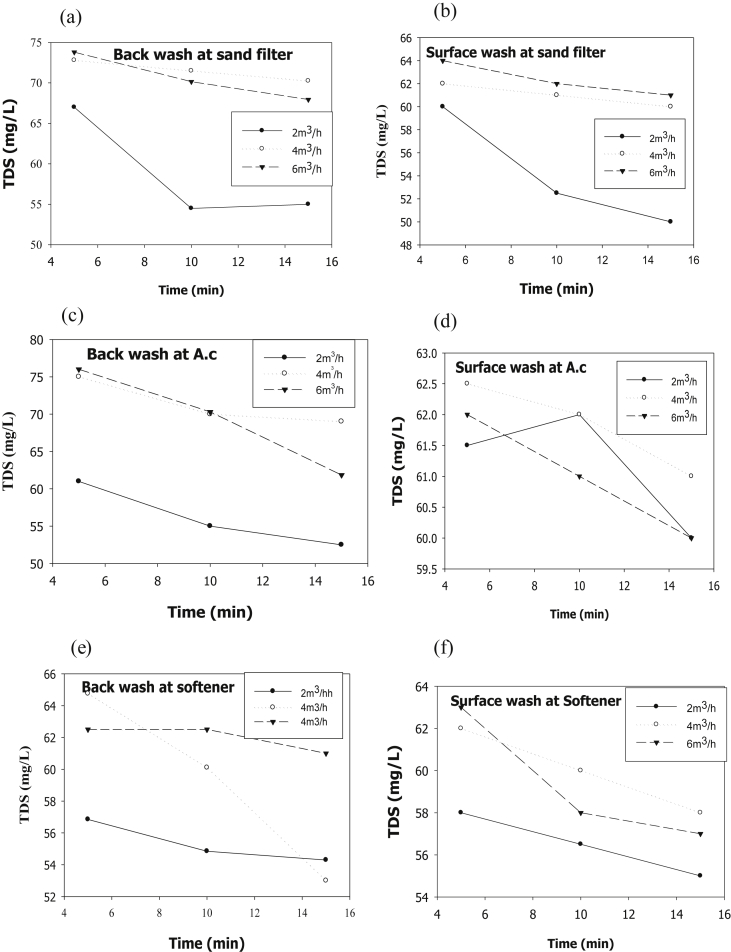
Total dissolved solid value at (a) Sand filter BW and (b) SW, (c) Activated Carbon at BW and (d) SW, (e) Ion-exchanger at BW and (f) SW.

**Fig. 6:**
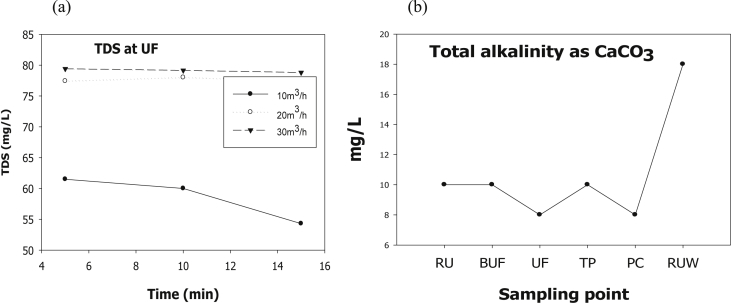
Total dissolved solid value (a) at UF and (b) Total alkalinity at all sampling points.

**Fig. 7:**
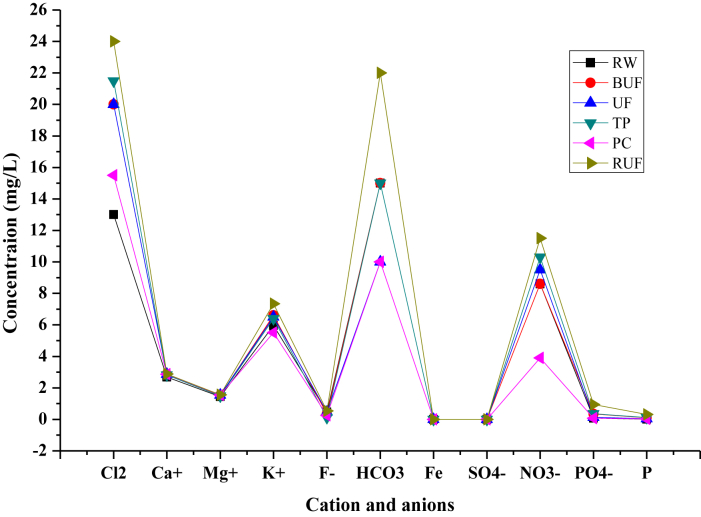
Total dissolved ion concentration at all sampling point.

**Fig. 8:**
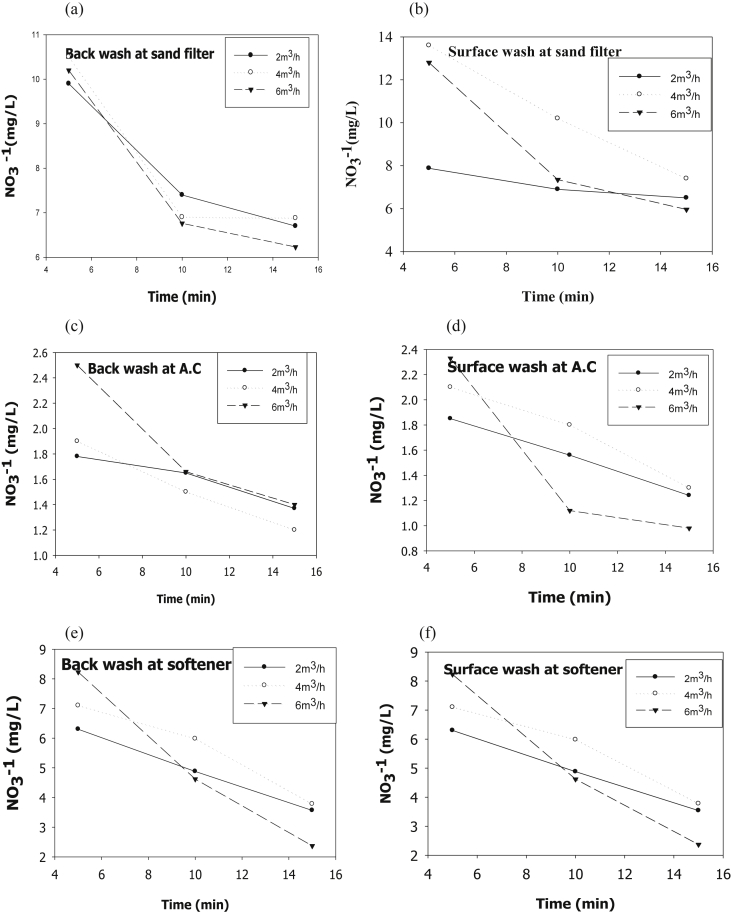
Total dissolved solid value at (a) Sand filter BW and (b) SW, (c) Activated Carbon at BW and (d) SW, (e) Ion-exchanger at BW and (f) SW.

**Fig. 9:**
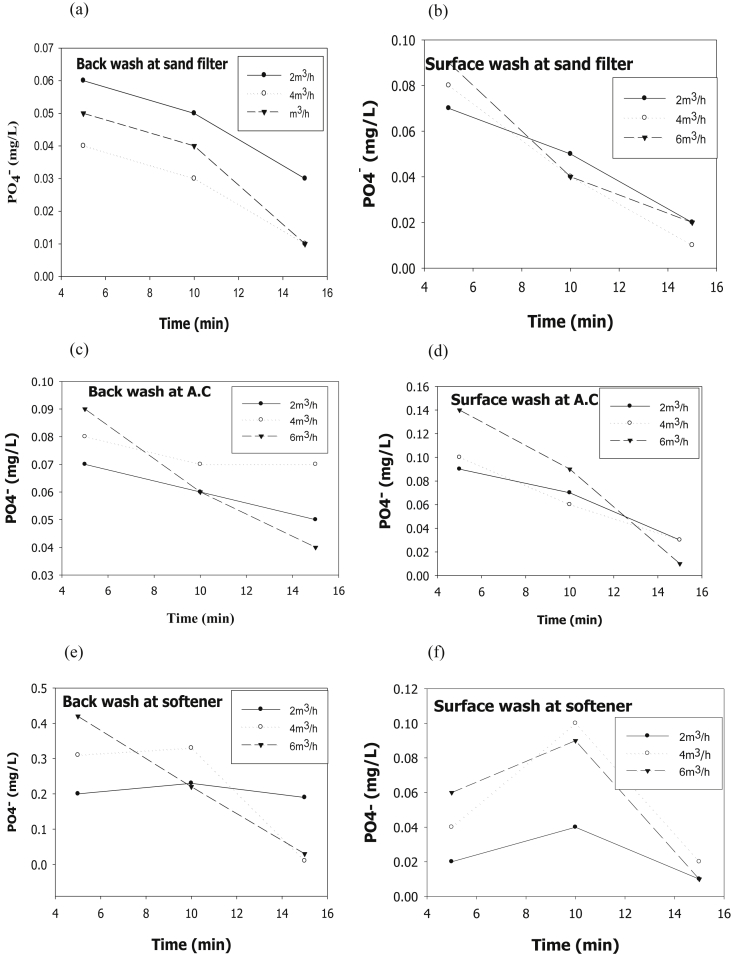
Phosphate value at (a) Sand filter BW and (b) SW, (c) Activated Carbon at BW and (d) SW, (e) Ion-exchanger at BW and (f) SW.

The evaluation of performance efficiency of the plant was undertaken in terms of effluent quality at each unit operation. Evaluation was based on the plant operation data such as discharge flow rate, washing time and sampling point. Daily variation of these parameters the process data are shown graphically Fig. 1 describes the pH values at various sampling points. Fig. 2 describes the turbidity value at different unit process with back wash and surface wash. Fig. 3 describes the total hardness of raw water at different seasonal and various sampling point. Fig. 4 displays the measure value of Electrical conductivity water at various unit filters. Fig. 5 describes the total value of dissolved solid value at each unit operation. Fig. 6 describes the total value of dissolved solid at ultra-filter and total alkalinity at different sampling points. Fig. 7 displays the total dissolved cation and anion concentration at all sampling point. Fig. 8 refers to nitrate value at all unit filtration and washing type. Fig. 9 describes the phosphate value at all unit operation. Table 1 describes the characteristics test value of Guna spring bottled water with standard limit from WHO and Ethiopian bottled drinking water specification. Table 2 refers to the result of Bacteriological analysis at different unit process discharged water during back and surface wash.

**Table 1:** Summary of characterized result Guna spring bottled water with its standard specification.

Substance/characteristics	Test method	Tested Result	Ethiopian bottled drinking water specification (ET:597,2001), maximum permissible level	WHO, Maximum permissible level
Calcium (mg/l)	ES ISO 7980	2.83	75	75
Chloride (mg/l)	ES ISO 9297	19.00	250	250
Fluoride (mg/l)	ES ISO10359-1	0.365	1.5	1.5
Iron (mg/l)	ES ISO 6332	0.00	0.3	0.3
Magnesium (mg/l)	ES ISO 7980	1.5166	50	50
Nitrate (mg/l)	ES ISO 7890-3	8.733	50	50
PH	ES ISO 10523	6.23	6.0 to 8.5	6.5–8.5
Phosphorus (mg/l)	ES ISO 6878	0.1050	0.1	0.1
Potassium (mg/l)	ES ISO 9964-2	6.388	50	50
sodium (mg/l)	ES ISO 9964-1	0.970	200	200
Sulfate (mg/l)	ES ISO 9280	0.0	250	250
TDS (mg/l)	ES 607	69.433	1000	1000
Total alkalinity (mg/l)	ES ISO 9963-1	10.66	200	200
Total hardness (mg/l)	ES 609	41.33	300	300
Turbidity (NTU)	ES ISO 7027	2.00	5.0	5.0
E.C (μS/cm)	ES ISO 7888	108.566	2000	2000

**Table 2:** Bacteriological analyzed at different unit process discharged water during BC and SW.

Sampling points	TC (MPN/100 ml)	FC(MPN/100 ml)	*E. coil* type1	Ethiopian BDWS	WHO Guidelines
Raw water	5	2	Absent	Absent	Absent
Sand filter	0	0	Absent	Absent	Absent
Activated carbon	0	0	Absent	Absent	Absent
softener	0	0	Absent	Absent	Absent
Ultra-filter	0	0	Absent	Absent	Absent
Collection tank	0	0	Absent	Absent	Absent
Reuse water	0	0	Absent	Absent	Absent

There are conventional methods for purification of water from ground and surface water for potable water includes Chemical precipitation, chemical oxidation and reduction, ion exchange filtration, sand filter, Electrochemical treatment, river osmosis, membrane technology and evaporation [4,5]. Recently years there is also an environmental ecofriendly and cost effective absorption method from agricultural materials [6] used as potential for low cost adsorbent in waste water treatment. Given the characteristics of raw discharged waste water and the requirement for reuse, the waste water usually requires some type of treatment before it is rendered fit for reuse as potable water. The complete treatment of waste water is brought by sequential combination of various physical unit operation, and chemical and biological unit process. Performance evaluation of the existing Guna treatment plant is required to assess the existing effluent quality and/or to meet higher treatment requirement and, to know about the treatment plant whether it is possible to handle higher hydraulic and organic loading. Performance appraisal practice of existing treatment plant units is effective in generation of additional data which also can be use in the improvement in the design procedure to be followed for design of these units.

## 2 Experimental design, materials, and methods

### 2.1 Material and chemicals

The data was conducted from Guna Spring Water Bottling Factory in Kimir Dingai Farta Woreda, Debre Tabor city which is located in South Gondar zone of the Amhara National Regional State of Ethiopia. Chemicals or medias were used for bacteriological analysis such as, nutrient agar to develop an aerobic plate count bacteria were used as a food, MacConkey broth used for presumptive confirming coli-form test in MPN dilution method as a food, Brilliant green bile broth (BGBB) was used to confirm total coli form present in the positive presumptive tests, EC broth was used to confirm fecal coli form present in the positive presumptive tests, Nutrient broth (NB) used to develop *E. coli* type1 present in the positive presumptive tests used as a food, and Kovacus reagent used to confirm the presence or absence of *E. coli* type1in the positive presumptive test confirming coil form test. Chemicals were used for physico-chemicals analysis such as, Nitra-ver5, Nitrate reagent powder pillow as determined nitrate level in the sample,Phos-ver3 (Ascorbic acid) reagent was determined phosphate level in the sample, DPD1 tablets were used to measure free chlorine residual, Silica oil to hide or remove the scratches on the vial during free chlorine residual and turbidity measurement, Al_2_(SO_4_)_3_ was used as a coagulant, hardness tablets also used to measure the total hardness in the sample, calcium hypochlorite was used as a disinfectant and sodium thiosulfate was used to neutralize the sample container for bacteriological analysis.

### 2.2 Preparation of media

35 g of Mac Conkey was dissolved in 1000 mL deionizer water using an Erlenmeyer flask, then mixed well by using hot plate with shaking and rotate it until completely dissolved and then observe a clear color on conical flask above the molten solution. Thereafter, sterilized in autoclave at 21 °C for 15 min, and then stored the molten solution at 44.5–50 °C in oven, and 24.64 g of nutrient broth was dissolved in 1000 mL deionizer water using a conical flask then mixed well by using hot with shaking and rotate it until completely dissolved and then observe a clear color on conical flask above the molten solution. Thereafter, sterilized in autoclave at 21 °C for 15 min to remove the contamination, and then stored the molten solution at 50 °C in oven.

## 3 Experimental design and descriptions

### 3.1 Bacteriological sampling

Sampling for bacteriological examination was carried out using sterile container of glass in 100 mL volume. Samples were preserved under low temperature of -4 °C before analysis done [1]. The time between sampling and analysis should not exceed 6 h**.** The bacteriological analysis was take place in the study factory and not exceeded 2 h after the sample collected.

### 3.2 Physical and chemical sampling

For physical and chemical analyzing, samples were stored in a clean polyethylene bottles at a low temperature in the sampling ice box. The sampling container was free from any contamination. The sample collection and analysis was taken six consecutive months from July up to December. At the selected sampling points within the design washing time and flow rate 1000 mL sample was taken in each unit process. Then after, the collected samples were stored in refrigerator until the analysis take place but taste, odor, color, turbidity, temperature, and pH were determined immediately on each unit operation or process sampling points where as the other tested parameter done in the laboratory after store the sample in a refrigerator [3].

Bacteriological analysis was carried out for indicator organism's i.e. total and fecal coli form and *E. coli* type1 by most probable number (MPN) method [2]. Calcium and Magnesium was determined by using AAS (NOVAA 400P). AAS is a technique for measuring quantities of chemical elements present in environmental samples by measuring the absorbed radiation. Iron, Sulfate, Chloride, Fluoride, total alkalinity, free chlorine residual, and turbidity were determined by using photometer. Nitrate and phosphate content were determined by using Spectrophotometer analysis. The Sodium and potassium content were determined by Flame photometer (FP 640) analysis at a maximum emission intensity of 589 nm and 766.5 nm respectively.
